# Metabolic modeling suggested noncanonical algal carbon concentrating mechanism in *Cyanidioschyzon merolae*

**DOI:** 10.1093/plphys/kiaf019

**Published:** 2025-01-16

**Authors:** Maneesh Lingwan

**Affiliations:** Plant Physiology, American Society of Plant Biologists, Rockville, MD 20855-2768, USA; Donald Danforth Plant Science Center, St. Louis, MO 63132, USA

Extremophilic organisms are fascinating due to their unique biology, which makes them valuable as functional model organisms and potential candidates for biotechnology applications. *Cyanidioschyzon merolae* is an extremophilic red microalga and one of the few organisms that rely on photosynthesis in high-temperature environments, where hot and acidic conditions limit the availability of inorganic carbon and challenge biological carbon fixation. Green algae grow in challenging environments by using a carbon-concentrating mechanism (CCM) involving a specialized pyrenoid structure. CCMs enhance photosynthetic efficiency by concentrating CO_2_ around Rubisco, providing adequate substrate for carbon fixation and inhibiting the competing oxygen-fixation reaction of Rubisco (Miyagishima and Tanaka 2021). Unlike green algae, the ability of the red algae *C. merolae* to assimilate CO_2_ efficiently is not well-characterized, and the presence and operation of its CCM is not unequivocal. For example, it lacks the anatomical structures associated with pyrenoid CCM organelles and cannot take up external bicarbonate. In such cases where biochemical knowledge is limited, mathematical and computational surrogate modeling approaches can help to understand possible cellular and molecular mechanisms in organisms and how they survive extreme environmental challenges (Fei et al. 2022; Steensma et al. 2023).

Recently in *Plant Physiology*, Steensma et al. (2024) demonstrated that the kinetics of *C. merolae* Rubisco are like other red algae and while they have a strong affinity for CO_2_, a weak affinity for O_2_, and a slow carboxylation rate, they still must employ a CCM based on measured CO_2_ assimilation rates. To further investigate how the *C. merolae* CCM may function, the authors constructed a mathematical model incorporating parameters aligned with existing literature and experimental measurements. The hypothetical CCM described in the model has a set of well-mixed compartments in which carbon diffuses into the cell as CO_2_, which is converted to bicarbonate in the cytosol by carbonic anhydrase (CAs) and transported to the chloroplast, where another CAs supplies CO_2_ to Rubisco. However, some reports suggested the potential effects of the concentric thylakoids in *C. merolae* (Fig.). CAs and bicarbonate transporters are potentially important components of known biophysical CCMs and are thus considered key elements in any CCM model (Beardall and Raven 2020).

**Figure.**
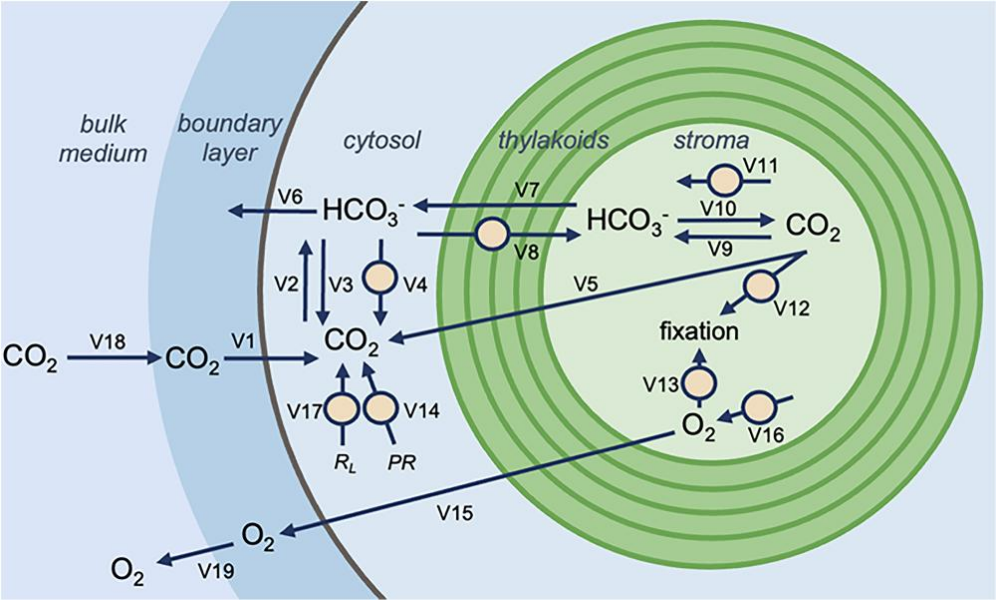
The model illustrates that molecule pools can occur in the boundary layer to form a series of concentric spherical well-mixed compartments in *Cyanidioschyzon merolae*. The external bulk medium is surrounded by a lipid bilayer as a boundary medium around the cell, followed by the cytosol, with concentric thylakoids and stromal space within the chloroplast. Arrows indicate the metabolic fluxes and pools of dissolved inorganic carbon systems are indicated by molecular formulas such as carbon dioxide (CO_2_), bicarbonate (HCO_3_^−^), and oxygen (O_2_). PR represents photorespiratory CO_2_ release, and R_L_ stands for respiration in the light. Small circles denote enzymatically catalyzed fluxes. A range of transport conditions addresses the number of molecules that cross between the cytosol and the stroma, and the energy cost of this transport was captured by varying membrane parameters (V) in compartments. For example, diffusion through lipid membranes represented as V1, V6, V5, V7, and V15 to estimate the conductivity of lipid membranes to the chemical species. Whereas spontaneous interconversion of CO_2_ and HCO_3_^−^ represented as in V2, V3, V9, and V10. Reprinted from Steensma et al. (2024).

In algal systems, growth under limited CO_2_ levels is supported by active bicarbonate uptake; however, CCM in *C. merolae* operates without any active uptake of bicarbonate from the environment. An additional constraint is that organisms like cyanobacteria enclose Rubisco in a protein shell called the carboxysome and in algae pyrenoid surrounded by a starch sheath that acts as a diffusion barrier for CO_2_. In contrast, there is no pyrenoid diffusion barrier in *C. merolae*, as neither a starch sheath nor an organized subcompartment for Rubisco has been reported in this species. So, Steensma et al. (2024) attempted to model the nature of the diffusion barrier surrounding Rubisco in *C. merolae*. They tested different parameterizations that provided quantitative support for a CCM where inorganic carbon enters the cell exclusively by passive CO_2_ diffusion into the cytosol, without specialized compartments, which authors named as “noncanonical” CCM due to its differences in structure and function from regular CCMs (Fig.) (Steensma et al. 2024). Previously, acidophile algae were reported to accumulate carbon through bicarbonate-trap or acid-loading mechanisms; this involves concentrating bicarbonate via enzymatic action and permeability to cell membranes (Curien et al. 2021; Fei et al. 2022).

To determine if the model results were the result of selecting a narrow subset of parameterizations of the model, the authors not only incorporated known physiological and enzymatic data into the parameterizations but also used a surrogate model developed using machine learning to explore the possible parameter space more fully. The model incorporated experimental data on gas exchange and Rubisco parameters, which are distributed throughout a series of concentric thylakoid membranes. This model setup explores whether these membranes, thought to be highly permeable to CO_2_, could influence carbon concentration or photosynthetic efficiency. Model outputs are consistent with other studies indicating that thylakoid membranes could impact inorganic carbon diffusion. Modeling suggests that *C. merolae* CCM may have a minimal mechanism that includes thylakoid membranes acting as diffusion barriers and concentric thylakoids do increase carbon concentration by providing a barrier to prevent CO_2_ leakage but increase the cost of bringing carbon into the stroma (Steensma et al. 2024). Previous models incorporating detailed chloroplast geometry demonstrate that efficient carbon capture can occur in a simplified scenario where Rubisco and CAs are distributed throughout a series of concentric thylakoid spheres (Fei et al. 2022). Additionally, the authors developed a method to assess the sensitivity to key parameters of interest in models. Robust parameter exploration and statistical analysis helped to identify the features that influenced the measures for functional CCM and efficient energy usage. Key parameters impacting functional outputs included cytosolic pH, the cost and kinetics of bicarbonate pumping, cell radius, carboxylation velocity, and CO_2_ membrane permeability. In summary, compartmental photosynthetic modeling could inform experiments and rational metabolic engineering to improve agricultural productivity and industrial advantage.

## Data Availability

No new data were generated or analyzed in support of this research.
